# High Heating Rates Affect Greatly the Inactivation Rate of *Escherichia coli*

**DOI:** 10.3389/fmicb.2016.01256

**Published:** 2016-08-11

**Authors:** Juan-Pablo Huertas, Arantxa Aznar, Arturo Esnoz, Pablo S. Fernández, Asunción Iguaz, Paula M. Periago, Alfredo Palop

**Affiliations:** ^1^Departamento de Ingeniería de Alimentos y del Equipamiento Agrícola, Escuela Técnica Superior de Ingeniería Agronómica, Universidad Politécnica de CartagenaCartagena, Spain; ^2^Unidad de Microbiología y Seguridad Alimentaria, Instituto de Biotecnología Vegetal, Universidad Politécnica de CartagenaCartagena, Spain

**Keywords:** heat resistance, heating rate, *Escherichia coli*, heat exchanger, thermoresistometer

## Abstract

Heat resistance of microorganisms can be affected by different influencing factors. Although, the effect of heating rates has been scarcely explored by the scientific community, recent researches have unraveled its important effect on the thermal resistance of different species of vegetative bacteria. Typically heating rates described in the literature ranged from 1 to 20°C/min but the impact of much higher heating rates is unclear. The aim of this research was to explore the effect of different heating rates, such as those currently achieved in the heat exchangers used in the food industry, on the heat resistance of *Escherichia coli*. A pilot plant tubular heat exchanger and a thermoresistometer Mastia were used for this purpose. Results showed that fast heating rates had a deep impact on the thermal resistance of *E. coli*. Heating rates between 20 and 50°C/min were achieved in the heat exchanger, which were much slower than those around 20°C/s achieved in the thermoresistometer. In all cases, these high heating rates led to higher inactivation than expected: in the heat exchanger, for all the experiments performed, when the observed inactivation had reached about seven log cycles, the predictions estimated about 1 log cycle of inactivation; in the thermoresistometer these differences between observed and predicted values were even more than 10 times higher, from 4.07 log cycles observed to 0.34 predicted at a flow rate of 70 mL/min and a maximum heating rate of 14.7°C/s. A quantification of the impact of the heating rates on the level of inactivation achieved was established. These results point out the important effect that the heating rate has on the thermal resistance of *E. coli*, with high heating rates resulting in an additional sensitization to heat and therefore an effective food safety strategy in terms of food processing.

## Introduction

Microbial heat resistance studies are necessary for the safe production of heat processed foods. The knowledge provided by these studies on microbial destruction kinetics and on the mechanisms of inactivation has allowed the design and development of safe processes, eliminating the risk of foodborne pathogen and spoilage microorganisms. Also, the correct application of thermal treatments results in avoiding overprocessing of food products.

Thermal resistance of microorganisms is affected by many different factors. Some of the most influencing factors are the water activity, nutrient content, pH of the heating medium, growth phase and growth temperature of the microbial culture, as well as the genus, species and even the strain within the same species. The research on these factors has been usually performed under isothermal treatment conditions. However, heat treatments applied in food industry comprise non-isothermal stages (corresponding to heating and cooling phases), which may be even more important than the isothermal stage (holding phase) in terms of inactivation of microorganisms.

One of the factors influencing heat resistance to which authors have paid less attention is the heating rate, probably because of the lack of appropriate equipment to measure this effect. This fact has led some authors to develop and use non-isothermal methods as an alternative to understand microbial inactivation kinetics under these heating conditions (Reichart, 1979; Periago et al., 1998; Fernández et al., 1999; Conesa et al., 2003; Hassani et al., 2005; Valdramidis et al., 2006; Van Derlinden et al., 2010; Esteban et al., 2013). Some of these researches (De Cordt et al., 1992; Periago et al., 1998) have shown that there are differences between the heat resistance values obtained under isothermal and non-isothermal heating conditions.

Many different heat resistance determination methods and instruments have been used (Stumbo, 1973; Brown and Ayres, 1982; Palop et al., 2012), each of them having their own advantages and drawbacks. In 2009 Conesa et al. built the thermoresistometer Mastia, where most advantages of the existing methods were incorporated. Its only limitations were that the maximum heating and cooling rates it was able to provide were about 35°C/min, and that it worked as a batch system. These heating and cooling rates were fast enough to mimic batch heating systems, such as retorts (Lewis, 2006), but did not achieve the faster heating rates reached at continuous heating systems, such as heat exchangers. Continuous processing minimizes the exposure time of food products at high temperatures because of the high heating and cooling rates reached on these systems, reducing the adverse effects of thermal treatments on food quality and also minimizing the processing times (Tucker et al., 2002). These limitations led Huertas et al. (2015) to build a pilot plant heat exchanger, in which it was possible to mimic in-flow processes, measure the temperature and take several samples along its pipelines, enabling to build survival curves at faster heating rates. However, this heat exchanger cannot reach very high heating rates, which in some processes, such as HTST or UHT systems could be almost instantaneous. This limitation is a hindrance on the exploration of the effect of heating rate on microbial heat inactivation. Still, the thermoresistometer Mastia can be used for continuous heating processes, in which much faster heating rates could be achieved.

The objectives of this research were to explore the effect of high heating rates on the thermal inactivation of *E. coli* and to evaluate the thermoresistometer as a continuous heating system.

## Materials and methods

### Microorganisms

*Escherichia coli* type strain (CECT 515) was provided by the Spanish Type Culture Collection (CECT). Cells were grown overnight at 37°C in tryptic soy broth (TSB; Scharlau Chemie) supplemented (w/v) with 0.6% yeast extract (YE; Scharlau Chemie), until the stationary phase of growth was reached.

### Heating medium

Citrate phosphate pH 7 McIlvaine buffer was prepared as described by Dawson et al. (1974) and was stored at 0–5°C until used.

### Determination of heat resistance in the heat exchanger

A pilot plant scale, double tube heat exchanger (Huertas et al., 2015) was used. The heating medium inoculated with the microorganism was pumped through the product pipe, as described by Huertas et al. (2015), into the system at different flow rates, 480 and 780 mL/min. The heat exchanger was programmed to raise the temperature of the product to 60 or 65°C. The sampling points located along the heating sections enabled to measure the temperature and to take samples for microbiological analysis along with heating. Three different maximum heating rates were reached: 21°C/min, with a flow rate of 480 mL/min and a final temperature of 60°C and 32°C/min, with a flow rate of 780 mL/min and a final temperature of 60°C and 50°C/min, with a flow rate of 780 mL/min and a final temperature of 65°C. The experiments were repeated at least three times.

### Determination of heat resistance in the thermoresistometer

The thermoresistometer Mastia (Conesa et al., 2009) was used in a continuous mode. This operating mode consisted in filling the vessel of the thermoresistometer with water and heating it at a preset temperature. Then, the process medium, already inoculated with the microorganisms, was circulated through the cooling system coil by means of a peristaltic pump (Selecta, Barcelona, Spain) at a controlled flow. In this way, the instrument works as a heat exchanger, with a constant temperature of the water used as heating fluid. The dimensions of the coil were 160 cm long (110 cm were immersed inside the thermoresistometer and 25 cm corresponded to each branch outside the instrument), 3.2 mm of inside diameter and a total volume of 12 mL. The coil was previously sterilized *in situ*. The input temperature of the microbial suspension, before pumping through the coil, was 20°C in all cases. The output temperature of the microbial suspension was continuously measured after passing through the coil, by means of a thermocouple located just after the 25 cm long output branch of the coil, well outside the vessel. After this probe, a short silicon tube was placed to enable sampling for microbiological counts. When the output temperature was constant the system was in steady state. Then, suspension samples were taken and quickly cooled at room temperature. In this way, very fast heating (estimated as described in Temperature Profile and Mean Residence Time Estimation) was achieved (up to 22.5°C/s), followed by a short holding period and an instantaneous cooling, similar to those on continuous food pasteurization treatments.

Experiments were carried out keeping a constant temperature of 65° or 70°C inside the vessel of the thermoresistometer, and passing the bacterial suspension through the coil at different speeds of the peristaltic pump. At least 3 samples were taken at each flow and the numbers of survivors were determined.

### Temperature profile and mean residence time estimation

The temperature profiles in the pilot plant heat exchanger were obtained with the Pt-100 temperature probes placed in each elbow, as described elsewhere (Huertas et al., 2015). Mean residence times were also calculated as described by Huertas et al. (2015).

The temperature profiles in the coil of the thermoresistometer were estimated according to Son and Singh (2002). The energy balance applied to a differential volume of the coil of length *dl* provides:

(1) $$\begin{array}{l} dq=U\cdot 2\pi \cdot r\cdot dl\cdot \left(T_{tr}-T\right)=m\cdot c_{p}\cdot dT \end{array}$$

Solving this differential equation, the same expression proposed by Deindoerfer and Humphrey (1959) was obtained:

(2) $$\begin{array}{l} \text{ln }\left(\frac{T-T_{tr}}{T_{i}-T_{tr}}\right)=-\frac{U\cdot 2\pi \cdot r}{m\cdot c_{p}}\cdot l \end{array}$$

where *T* is the temperature of bacterial suspension inside the coil at a distance *l* from the inlet (°C), *T*_*tr*_ is the thermoresistometer temperature (°C), *T*_*i*_ is the temperature of bacterial suspension at coil inlet (°C), *U* is the overall heat transfer coefficient (W/m^2^ × °C), *r* is the coil internal radius (m), *l* is the distance from the inlet of the coil (m), *m* is the mass flow rate (kg/s), *c*_*p*_ is the specific heat capacity of the suspension (J/kg × °C).

The overall heat transfer coefficient (*U*) was estimated according to Dichfield et al. (2006). This value was used to calculate the temperature profile inside the coil.

A small decay in the temperature corresponding to the output branch of the coil, outside the instrument, was observed at flows slower than 100 mL/min, even isolating the output coil pipe. This small decay was estimated as previously stated, with Equation (2).

Mean residence times in the coil were calculated for each flow and are shown in Tables 1, 2.

**Table 1:** **Mean residence time, maximum heating rate, outlet temperature and number of log cycles inactivated in the thermoresistometer under a constant temperature of 65°C at different flows vs. their corresponding predicted inactivation values**.

**Flow (mL/min)**	**Mean residence time (s)**	**Maximum heating rate (°C/s)**	**Outlet temperature (°C)**	**Predicted inactivation (log cycles)**	**Observed inactivation (log cycles) ± sd**
70	10.3	14.7	61.1	0.34	4.07 ± 0.17
77	9.4	15.4	61.2	0.27	3.12 ± 1.00
85	8.5	15.8	61.1	0.21	1.34 ± 0.34
95	7.6	17.1	60.9	0.17	0.31 ± 0.01
106	6.8	17.8	60.9	0.13	0.27 ± 0.08

**Table 2:** **Mean residence time, maximum heating rate, outlet temperature and number of log cycles inactivated in the thermoresistometer under a constant temperature of 70°C at different flows vs. their corresponding predicted inactivation values**.

**Flow (mL/min)**	**Mean residence time (s)**	**Maximum heating rate (°C/s)**	**Outlet temperature (°C)**	**Predicted inactivation (log cycles)**	**Observed inactivation (log cycles) ± sd**
95	7.6	17.3	64.7	0.84	3.82 ± 0.62
112	6.4	18.3	64.1	0.53	3.71 ± 0.78
133	5.4	20.4	63.4	0.31	2.78 ± 0.60
158	4.6	21.5	62.5	0.17	1.36 ± 0.01
185	3.9	22.5	61.1	0.14	0.27 ± 0.08

### Enumeration of survivors

Viable counts were based on duplicate counts, from appropriate dilutions, in tryptic soy agar (TSA; Scharlau Chemie)+ 0.6% YE. The plates were incubated for 24 h at 37°C. Preliminary experiments showed that longer incubation times did not modify plate counts.

### Data analysis

Experimental data in the present research were obtained under non-isothermal conditions. These experimental data were contrasted against survivor numbers predicted from *D*_*T*_ and *z* values obtained under isothermal conditions in a previous study (Conesa et al., 2009). In that research, *D*_*T*_ values were calculated from the slope of the regression line of survival curves as given by the Bigelow model (Equation 3):

(3) $$\begin{array}{l} LogN_{t}=logN_{0}-\frac{t}{D_{T}} \end{array}$$

where *N*_*t*_ is number of microorganisms at time *t* and *N*_0_ is the initial number of microorganisms.

To predict the number of survivors in the present research, a rate model derived from the Bigelow model (Equation 3), representing the momentary time-dependent isothermal logarithmic inactivation rate was used. This rate model considers the non-isothermal treatments as composed of successive isothermal treatments of very short (differential) duration, each one at a different temperature, and hence can be written as an ordinary differential equation as given by Equation (4):

(4) $$\begin{array}{l} \frac{dlogN}{dt}=\frac{-1}{D_{T}} \end{array}$$

with the initial condition *N(0)* = *N*_0_.

The calculation of the *D* values for each of these different temperatures was based on the dependence of *D* with respect to temperature, which can be described with the classic Bigelow model as given by Equation (5):

(5) $$\begin{array}{l} D\left(T\right)=\frac{D_{T_{ref}}}{10^{\frac{T-T_{ref}}{z}}} \end{array}$$

where *D*_*Tref*_ is the *D(T)* value at the reference temperature (*T*_*ref*_), and *z* is the number of degrees Celsius change of temperature required to achieve a tenfold change in *D*-value.

Significant differences between counts of the sample replicates and the experiment repetitions were analyzed by ANOVA test (Statgraphics 5.1 plus, Manugistics Corp., Rockville, MD, USA) at the 95 % confidence level.

## Results

Thermal resistance characterization of *E. coli* CECT 515 under isothermal heating conditions in pH 7 McIlvaine buffer was taken from a previous study. An average D_60_ value of 0.38 min and a *z*-value of 4.7°C were obtained (Conesa et al., 2009), and were used in this research to predict the inactivation under non-isothermal heating conditions.

### Heat resistance in the heat exchanger

**Figure 1:**
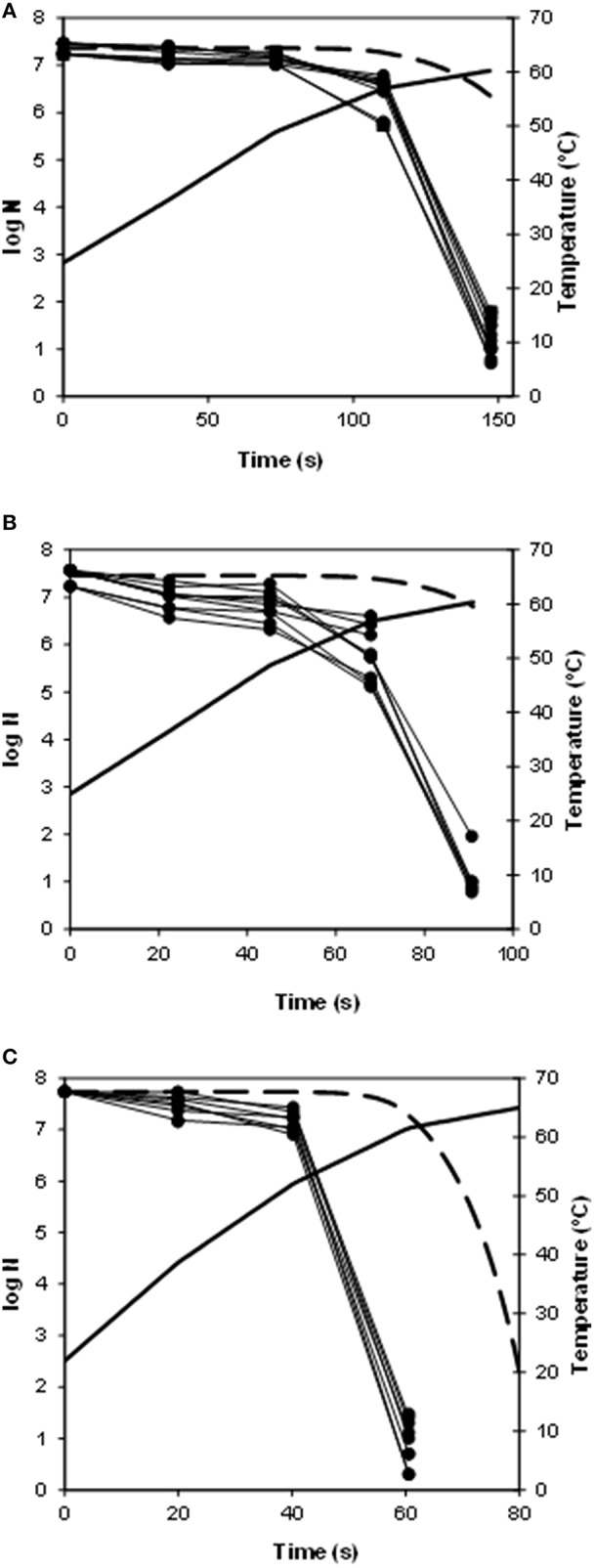
**Survival curves of ***Escherichia coli*** in flow at different heating rates on the pilot plant heat exchanger, together with the predicted inactivation (- -; thick line), and the temperature profile (^**__**^; thick line). (A)** maximum heating rate of 21°C/min; **(B)** maximum heating rate of 32°C/min; **(C)** maximum heating rate of 50°C/min.

Figure 1 shows the heating profiles of the different experiments performed in the heat exchanger, together with the observed and predicted inactivation data. Since this heat exchanger is provided with temperature sensors and sampling points along the heating section (Huertas et al., 2015), it was possible to determine the temperature profiles and to obtain samples during the entire thermal process, which permits to follow the inactivation of the microorganisms along the heat exchanger and to observe the effect of the different heating rates on this inactivation. The maximum heating rate obtained at the beginning of the experiment was 50°C/min, for a flow rate of 780 mL/min and a final temperature of 65°C (Figure 1C) and the minimum was 21°C/min, for a flow rate of 480 mL/min and a final temperature of 60°C (Figure 1A). As it can be observed in Figure 1, the higher the heating rate, the earlier the inactivation of the bacterial cells starts: for the treatments with a maximum heating rate of 50°C/min (Figure 1C), 32°C/min (Figure 1B), and 21°C/min (Figure 1A), about seven log cycles were inactivated in 60, 90, and 150 s respectively. These differences in time to inactivate were somehow expected since at higher heating rates, lethal temperatures are reached faster than at lower heating rates. Still, it is noteworthy that the inactivation obtained experimentally was much higher than the one predicted by using the isothermal data, under all the experimental conditions (Figure 1): in all cases, when the observed inactivation had reached about seven log cycles, the predictions were estimating about 1 log cycle of inactivation. Hence, all these non-isothermal heating profiles were more lethal than expected or, in other words, predictions were well within the fail-safe side in all cases.

### Heat resistance in the thermoresistometer

**Figure 2:**
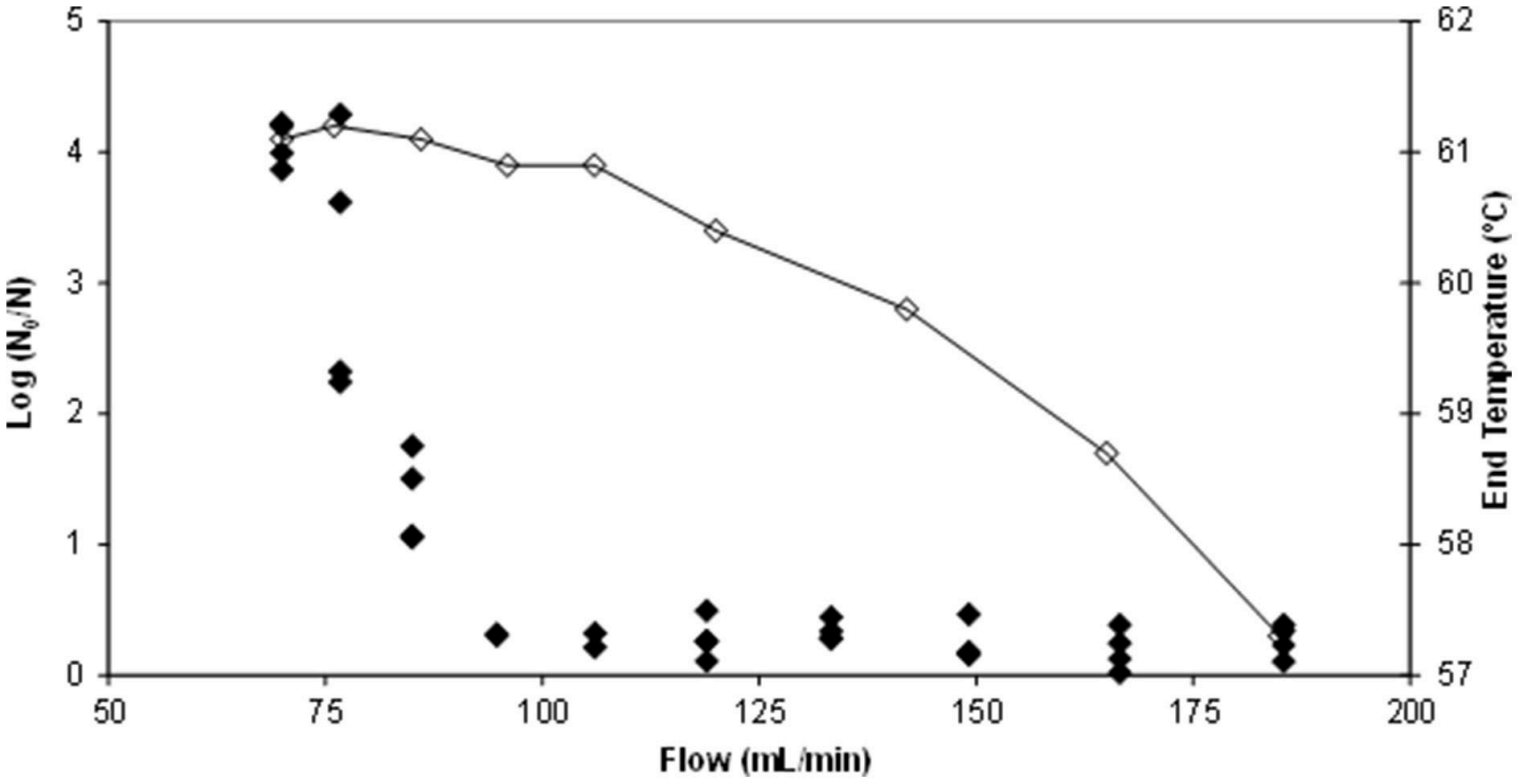
**Temperature measured (⋄) at the end of the coil for each flow (mL/min) when operating the thermoresistometer in continuous mode and the corresponding number of log cycles inactivated (♦)**.

**Figure 3:**
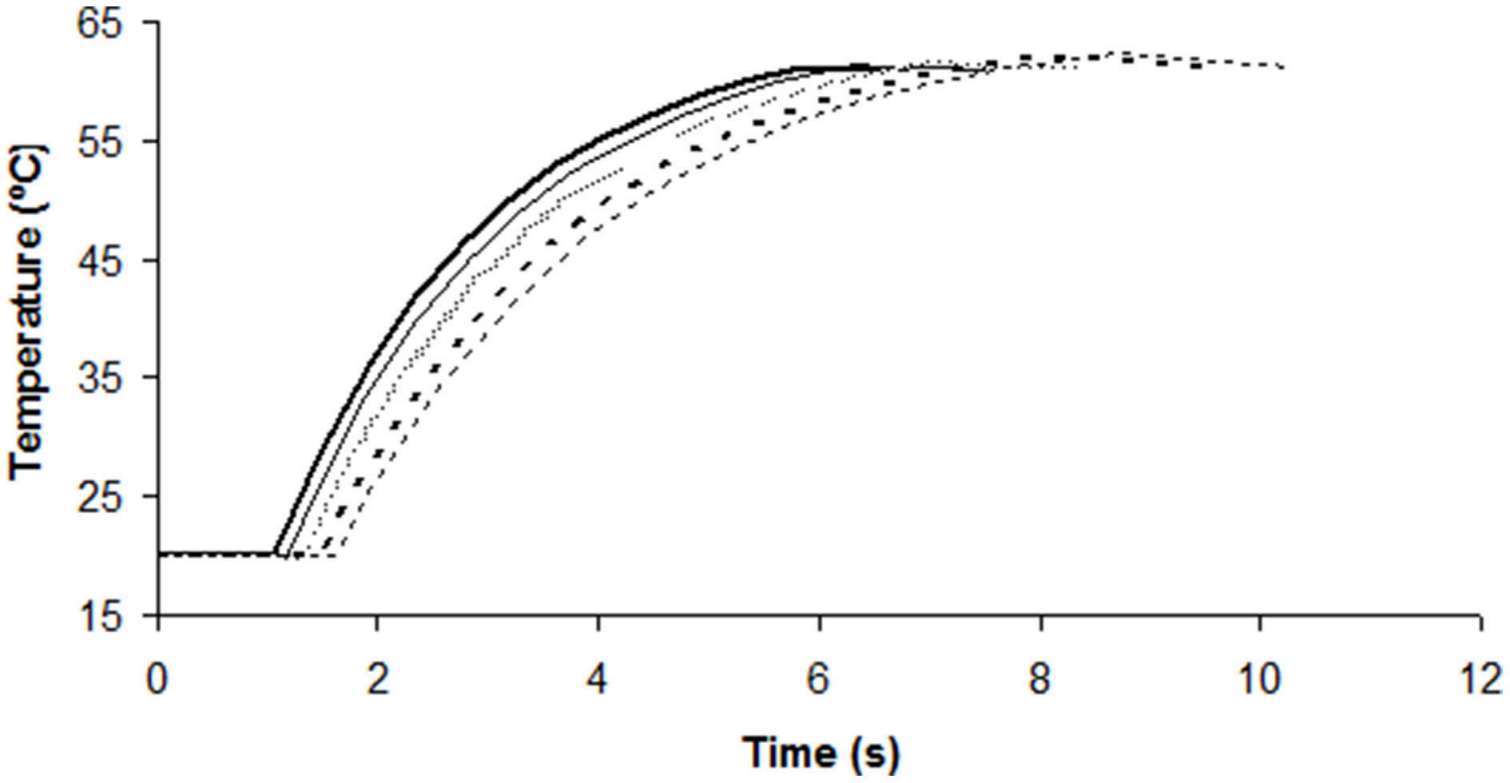
**Estimated temperature evolution inside the coil for flows of 70 (- -; thin lines), 77 (- -; thick lines), 85 (^**…**^), 95 (^**__**^; thin lines), and 106 (^**__**^; thick lines) mL/min, under a constant temperature of the thermoresistometer of 65 °C**.

Figure 2 shows the output temperature (after passing through the thermoresistometer preheated at 65°C) for each flow and the corresponding number of survivors. Figure 3 depicts the evolution of temperatures inside the coil for flows of 70, 77, 85, 95, and 106 mL/min, estimated by means of Equation (2), and considering the mean residence time, when the thermoresistometer was preheated at 65°C. For flows lower than 100 mL/min the temperature decay in the output pipe of the coil, outside the instrument, was also estimated with Equation (2). At flows faster than 106 mL/min, residence times of the suspension inside the whole coil were lower than 6 s and the temperature decay in the output pipe was negligible. At flows faster than 95 mL/min, residence times were too short to reach the treatment temperature in the coil an almost no population reduction was observed, in spite of the fast heating rates (Figure 2). At flows between 70 and 95 mL/min, the slower the flow, the more heat inactivation was observed, since longer residence times were achieved while the output temperature was very similar in all cases (Figure 2).

Similar results were obtained when the thermoresistometer was preheated at 70°C (data not shown). However, since the temperature reached inside the coil was higher, faster flows and shorter residence times were needed to achieve similar levels of inactivation.

The estimation of temperatures along the coil by means of Equation (2) enabled to predict the microbial inactivation reached by these treatments (based on heat resistance data obtained under isothermal heating by Conesa et al. (2009) together with Equations 4, 5), which are represented in Tables 1, 2, together with the observed values of log cycles of inactivated bacteria. Flow rates, mean residence times, outlet temperatures and maximum heating rates reached by these treatments are also included in these tables. Heating rates between 14.7 and 22.5°C/s (i.e., up to 1350°C/min) were obtained at the beginning of these treatments (Tables 1, 2), which are much higher than those achieved in the heat exchanger.

Table 1 shows the results obtained for several flows when the constant temperature inside the vessel was 65°C (those corresponding to the experiment depicted in Figures 2, 3). At a flow rate of 106 mL/min scarcely 0.27 log cycles were inactivated (Table 1), probably because the mean residence time (6.8 s) in the coil was too short to achieve higher levels of inactivation, even when lethal temperatures (close to 61°C; Figure 2) were reached. Actually, only 0.13 log cycles of inactivation were predicted for this thermal treatment (Table 1), but significant differences were found at this flow rate between observed and predicted values. At faster flow rates, similar low inactivation levels observed were shown (Figure 2), but no significant differences were found between observed and predicted values, because of the broader dispersion of the microbial counts at these faster flow rates. However, at slower flow rates, enhanced inactivation was observed, and significant differences between predicted and observed values increased at low flow rates. Hence, at a flow rate of 70 mL/min, the observed inactivation was more than 10 times greater than the predicted (4.07 log cycles inactivation observed; 0.34 log cycles inactivation predicted; Table 1). At slower flow rates, where low inactivation was also predicted, complete microbial inactivation was reached (data not shown).

Table 2 presents the results obtained for a constant temperature inside the vessel of 70°C. Again, slower flow rates led to higher levels of inactivation, although in this case, the differences between observed and predicted values were not as big as for a constant temperature inside the vessel of 65°C. At flow rates slower than 95 mL/min, complete inactivation was reached (data not shown). The maximum difference between observed and predicted values was of about 9 times, with a flow of 133 mL/min (2.78 log cycles inactivation observed; 0.31 log cycles inactivation predicted; Table 2). Anyhow, and similarly to the results obtained in the heat exchanger (Figure 1), all the experimental inactivation values were significantly higher than their corresponding predictions, which means much higher inactivation than what could be expected from isothermal inactivation kinetics data and an additional safety measure because the predictions are on the “fail safe” side.

## Discussion

When applying continuous treatments in the food industry, such as those achieved in heat exchangers, heating and cooling rates are fast, much faster than those provided with the thermoresistometer Mastia (up to 35°C/min; Conesa et al., 2009). Experiments performed in the pilot plant heat exchanger reached heating rates as high as 50°C/min (Figure 1). Inactivation higher than expected from the isothermal data was achieved under all the experimental conditions (Figure 1). Deviations were particularly important at the late heating times. At these heating times, the temperatures reached (about 60°C in all cases) were lethal, but the previous thermal profile with an initial high heating rate, enhanced the lethality, leading to several extra log cycles of inactivation. These results prove that previous hypothesis regarding the more lethal effect of high heating rates than slow heating rates on the thermal resistance of this microorganism were correct (Conesa et al., 2009). These results are also in agreement with those obtained for other vegetative microorganisms, such as *Salmonella* or *Staphylococcus aureus* in this heat exchanger under similar treatment conditions (Huertas et al., 2015). Still, this pilot plant heat exchanger was not able to achieve the almost instantaneous heating rates that can be obtained under HTST and UHT treatments currently applied in the food industry. In order to overcome this limitation, the thermoresistometer Mastia was used in a continuous mode, using the coil as a heat exchanger, which enables to reach heating rates as high as 22.5°C/s. Using the instrument in this mode, the effect of very fast heating on the heat resistance of *E. coli* vegetative cells was investigated. The results of these experiments further confirmed the effect of the heating rates obtained in the heat exchanger.

In all cases, both for experiments performed in the heat exchanger and in the coil of the thermoresistometer, observed inactivation values were higher than predicted. When trying to look for a correlation between the initial heating rate of these experiments and the “over-inactivation” reached, several difficulties raised: the higher initial heating rates are linked to the shorter mean residence times, and consequently to the lower predicted inactivation values (see Tables 1, 2), so there were no similar treatments (in terms of predicted inactivation) with different heating rates. Also, the variability associated with bacterial counts, which is shown through the standard deviation values of Tables 1, 2 or through the data points shown in Figures 1, 2, hampers this comparison. Still, the only case with a similar predicted inactivation value (0.17 log cycles) leads to 1.36 log cycles inactivation when the initial heating rate was 21.5°C/s and the target temperature was 70°C (Table 2), and to only 0.31 log cycles inactivation when the initial heating rate is of 17.1°C/s and a target temperature of 65°C (Table 1), which would point out to this correlation between the heating rate and the inactivation reached.

These results reveal clearly that fast heating is much more efficient than isothermal treatments in killing *E. coli* vegetative cells. Previous research on the effect of heating rates on this same strain of *E. coli* showed that when cells were exposed to non-isothermal treatments at slow heating rates (2°C /min), their heat resistance was increased (Conesa et al., 2009), leading to less inactivation than expected. In this same research, it was shown that heating rates as high as 10°C/min led to opposite results, showing more inactivation than expected. It was hypothesized that at 2°C/min, some heat stress response could be induced, leading to an adaptation to heat, which would be absent at 10°C/min (Conesa et al., 2009).

The literature on the effect of heating rates on the heat resistance of vegetative cells is scarce and only explores the effect of heating rates as high as 10°C/min. The observed effects depend on the bacterial genus and on the heating rate value. Some bacterial genera became more heat resistant under high heating rates. For example, Hassani et al. (2006) showed that *S. aureus* exhibited higher thermal resistance at higher heating rates (up to 9°C/min), while others turned out to be more resistant at slow heating rates (Humphrey et al., 1993; Stephens et al., 1994; Morozov et al., 1997; Hassani et al., 2005; Valdramidis et al., 2006; Hassani et al., 2007). Also, Mañas et al. (2003) found no influence of the heating rate (between 0.5 and 4°C/min) on the thermal resistance of *Salmonella* Senftenberg 775 W, which, on the other hand, is an exceptionally heat resistant strain. These different behaviors could be explained in terms of genus, species or even strain variability. Even our research was performed with one only strain of *E. coli*. Still, all these researches were performed under heating rates much lower than the ones used in the present manuscript. During the first part of non-isothermal heating bacterial cells are exposed to non-lethal temperatures and it has been suggested that this exposure could entail an enhance on their heat resistance, similar to that observed when cells are exposed to isothermal treatments at sub-lethal temperatures, which act as a heat shock (Stephens et al., 1994; Mañas et al., 2003; Hassani et al., 2005; Valdramidis et al., 2006; Corradini and Peleg, 2007; Sergelidis and Abrahim, 2009; Van Derlinden et al., 2010), probably through the induction of heat shock protein (HSP) expression (Periago et al., 2002). These HSPs may be induced very rapidly (Allan et al., 1988; Yura et al., 2002). However, heating rates as fast as those that take place in heat exchangers are probably too fast to allow HSP synthesis.

Actually, the fastest heating rates are probably reached in the so-called isothermal heat resistance determination experiments performed in the microbiology labs, in which the microbial suspension is suddenly heated up to the treatment temperature. These experiments are then used to set the heat resistance of the microorganisms. For example, the thermal resistance of *E. coli* (*D*_60_ = 0.38 min; *z* = 4.7°C) was calculated inoculating 0.2 mL of the microbial suspension kept at room temperature into approx. 400 mL of the heating medium preheated at different treatment temperatures (Conesa et al., 2009), so, if this instantaneous heating (from room to treatment temperature) has any effect on the thermal resistance of the microorganism, it would be masked by the whole isothermal experiment and would be already taken into account when calculating microbial heat resistance. If this is the case, heat sensitization observed under the very high heating rates of about 20°C/s reached in the coil of the thermoresistometer in this research would be somehow unexpected, unless there is a difference between these very high heating rates and the instantaneous heating of isothermal experiments. If such difference exists, under very high heating rates there would be an effect of the heating rate and under instantaneous heating there would be no effect. Further research on high heating rates and instantaneous heating should be performed to unravel this hypothetic effect.

During the non-isothermal phases of treatment different phenomena may take place, which may affect heat resistance in some way. These phenomena and their effect on heat resistance should be considered when calculating the heat treatments to be applied in the food industry. The continuous operating mode of the thermoresistometer Mastia enables to determine the effect of high heating rates on bacterial cells, helping to understand the behavior and response of microorganisms to thermal treatments currently applied in the food industry. The results obtained in this research, as well as other studies from the scientific literature on other microorganisms of interest, could help to set more accurately the thermal treatment parameters. These proper settings would lead food industry to provide foods of better nutritional and sensorial quality and to save energy costs, while maintaining high standards of food safety.

## Conclusions

The heating rate plays an important role on the heat inactivation of microorganisms, when they are exposed to non-isothermal heat treatments. This factor has been usually omitted by authors when estimating microbial heat resistance. Heat resistance of *E. coli* vegetative cells was much lower than expected under high heating rates. Therefore, estimation of heat treatments based on isothermal *D* and *z* values may not provide a realistic estimation (although it falls on the fail-safe area) of the level of inactivation achieved when applying processing technologies, such as heat exchangers. Further research is required to quantify and understand this effect.

## Author contributions

Conceived and designed the experiments: AE, PF, AP. Performed the experiments: JH. Analyzed and interpreted the data: JH, AA, AE, AI, PP, AP. Drafted and revised the manuscript: JH, AA, AE, PF, AI, PP, AP.

## Funding

This research was financially supported by the Ministry of Economy and Competitiveness of the Spanish Government and European Regional Development Fund (ERDF) through project AGL2013-48993-C2-1-R.

### Conflict of interest statement

The authors declare that the research was conducted in the absence of any commercial or financial relationships that could be construed as a potential conflict of interest.
